# 

**Published:** 1874-10-01

**Authors:** 


No. XIX. CHICAGO, OCTOBER 1, 1874. Vol. XV.

### THE
### Medical Examiner.

A

### *Semi-Monthly Journal of Medical Sciences.*

*TERMS.—Yearly Subscriptions, Three Dollars, in advance. Single Number Fifteen Cents. All Communications should be addressed to Publishers* MEDICAL EXAMINER, 65 *Randolph st., cor. State, Chicago.*

